# An insight into the salivary gland and fat body transcriptome of *Panstrongylus lignarius* (Hemiptera: Heteroptera), the main vector of Chagas disease in Peru

**DOI:** 10.1371/journal.pntd.0006243

**Published:** 2018-02-20

**Authors:** Jessica C. Nevoa, Maria T. Mendes, Marcos V. da Silva, Siomar C. Soares, Carlo J. F. Oliveira, José M. C. Ribeiro

**Affiliations:** 1 Institute of Natural and Biological Sciences, Laboratory of Immunology, Federal University of Triângulo Mineiro, Uberaba, Minas Gerais, Brazil; 2 University of Texas at El Paso, El Paso, Texas, United States of America; 3 National Institute of Allergy and Infectious Diseases (NIAID), Laboratory of Malaria and Vector Research (LMVR), Rockville, Maryland, United States of America; University of Cincinnati, UNITED STATES

## Abstract

Triatomines are hematophagous arthropod vectors of *Trypanosoma cruzi*, the causative agent of Chagas Disease. *Panstrongylus lignarius*, also known as *Panstrongylus herreri*, is considered one of the most versatile triatomines because it can parasitize different hosts, it is found in different habitats and countries, it has sylvatic, peridomestic and domestic behavior and it is a very important vector of Chagas disease, especially in Peru. Molecules produced and secreted by salivary glands and fat body are considered of important adaptational value for triatomines because, among other functions, they subvert the host haemostatic, inflammatory and immune systems and detoxify or protect them against environmental aggressors. In this context, the elucidation of the molecules produced by these tissues is highly valuable to understanding the ability of this species to adapt and transmit pathogens. Here, we use high-throughput sequencing techniques to assemble and describe the coding sequences resulting from the transcriptome of the fat body and salivary glands of *P*. *lignarius*. The final assembly of both transcriptomes together resulted in a total of 11,507 coding sequences (CDS), which were mapped from a total of 164,676,091 reads. The CDS were subdivided according to their 10 folds overexpression on salivary glands (513 CDS) or fat body (2073 CDS). Among the families of proteins found in the salivary glands, lipocalins were the most abundant. Other ubiquitous families of proteins present in other sialomes were also present in *P*. *lignarius*, including serine protease inhibitors, apyrase and antigen-5. The unique transcriptome of fat body showed proteins related to the metabolic function of this organ. Remarkably, nearly 20% of all reads mapped to transcripts coded by Triatoma virus. The data presented in this study improve the understanding on triatomines’ salivary glands and fat body function and reveal important molecules used in the interplay between vectors and vertebrate hosts.

## Author summary

This work describes an in-depth salivary and fat body transcriptome from the triatomine *P*. *lignarius*, the main vector of Chagas disease in Peru. Following “de novo” assembly of over 160 million reads, 11 thousand coding sequences were obtained, 9,013 of which were submitted to GenBank. Those over expressed in the salivary glands or fat bodies were identified, leading to a detailed insight of the specificities of each organ. This work will serve future scientists not only for its increased knowledge on triatomine proteins, but also for its public protein sequences that will serve scientists as a target database in mass spectrometry experiments aiming at the identification of P. lignarius salivary pharmacological peptides or immunological targets of vector exposure.

## Introduction

*Panstrongylus lignarius*, also known as *Panstrongylus herreri* (WALKER, 1873) [1], is a triatomine species found in tropical and subtropical forests of South American countries including Peru, Ecuador, Colombia, Guyana, Suriname, Venezuela and Brazil [2, 3]. This species presents sylvatic behavior in the Amazon basin [4, 5] and peridomestic and domestic behavior in Peru [6]. Concerning its medical importance, this species is strongly synanthropic and is considered the major vector of Chagas disease in Peru [6, 7]. Among the triatomines of the genus *Panstrongylus*, the species *P*. *lignarius* is notable because, among other characteristics, it is capable of parasitizing different species of animals including marsupials, rabbits, spiny rats, anteaters, bats, chickens, toucans and pigeons [3]. In the Amazon region located in Peru, a considerable amount of the triatomines of this species are naturally infected with *Trypanosoma cruzi* (62.4%), and among those with identified food sources, 18.2% have been fed with human blood [8].

Among the mechanisms related to triatomine’s adaptation, it has been suggested that their saliva, which is inoculated during hematophagy, is crucial for the parasitism process and pathogen transmission. Indeed, the saliva of hematophagous arthropods, including triatomines, has inhibitory molecules of different defense mechanisms including platelet aggregation, inflammation, vasoconstriction, blood coagulation, and immune responses, which has been demonstrated to facilitate hematophagy and transmission of disease-causing agents [9].

In addition to saliva, molecules produced by the fat body from hematophagous arthropods have a substantial role in the detoxification of heme from blood, in developmental regulation and in the production of antimicrobial peptides and immunity [10–12]. Beyond these functions, the fat body is a multifunctional organ that has a pivotal role in nutrient and energy storage, in the synthesis of biomolecules and the whole metabolism [13]. It acts as a storage of energetic sources, important for the metamorphosis, egg maturation, reproduction and to survive long starvation periods. The fat body synthesizes and releases peptides, carbohydrates and lipids according to the metabolic needs and hormonal regulation [14].

It has been demonstrated through proteomic studies that triatomines of the *P*. *lignarius* species present a large number of bioactive molecules, but these molecules have a high interspecific functional biodiversity when compared to the molecules of the species *Triatoma lecticularia* and *Rhodnius prolixus* [15]. It has also been recently described that salivary molecules of *P*. *lignarius*, when compared to saliva of triatomines of the genus *Triatoma*, *Meccus* and *Rhodnius* have a remarkable ability to modulate dendritic cells and facilitate their invasion by *T*. *cruzi* [16].

The isolation and characterization of bioactive molecules in different tissues of blood-feeding insects has grown significantly in recent years and this scenario is mainly due to high-throughput sequencing techniques associated with bioinformatic tools. Different databases searches reveal genomes and sialomes of hematophagous arthropods such as ticks, mosquitoes and triatomines [17–24]. Here, we use high-throughput sequencing techniques to assemble and describe the coding sequences derived from a transcriptome of salivary glands and fat body of *P*. *lignarius*.

## Material and methods

### Ethics statement

The experiments were approved by the Institutional Animal Care and Use Committee—CEUA (protocol numbers 220 and 320).

### Insects

*P*. *lignarius* was obtained from the insectary of the Universidade Federal do Triângulo Mineiro, Uberaba, Minas Gerais, Brazil. The colonies were maintained in cylindrical recipients and fed weekly on chickens. The experiments were approved by the Institutional Animal Care and Use Committee—CEUA (protocol numbers 220 and 320). Fed adults, including 7 female and 7 male insects were used to collect salivary glands (SG) and fat body (FB). One couple was dissected every other day for 14 days. The SG and FB were stored in 200 μl and 400 μl of RNA later (Qiagen, Valencia, CA) respectively, at 4°C for 48 hours and then maintained at -80°C until the day of shipping. The samples from the 14 days were pooled together and used for qRT-PCR or sent lyophilized to NIH Intramural Sequencing Center (5625 Fishers Lane—Rockville, MD 20852).

### Sequencing

All procedures, including RNA extraction, libraries construction and sequencing were performed as previously described [23], with modifications. Briefly, RNA from each sample was collected using the Micro FastTrack-mRNA isolation kit (Invitrogen, Grand Island, NY) according to the manufacturer’s protocol. Following the isolation, total RNA integrity was checked using the BioAnalyser instrument (Agilent Technologies, Santa Clara, CA). The construction of mRNA libraries and sequencing were done at the NIH Intramural Sequencing Center. The fragments of cDNAs were made using a Covaris E210 (Covaris, Woburn, MA) and the libraries of SG and FB were constructed separately using the TruSeq RNA sample prep kit, v. 2 (Illumina Inc., San Diego, CA). Both libraries were amplified using eight cycles to minimize the risk of over-amplification. The sequencing of SG and FB were performed on a HiSeq 2000 (Illumina) with v. 3 flow cells and sequencing reagents. A paired-end protocol was used.

### Bioinformatics

Raw data were processed using RTA 1.12.4.2 and CASAVA 1.8.2. The reads were trimmed of low quality regions, and only those with an average Illumina quality of 20 or more were used. Afterwards, they were assembled using ABySS software (Genome Sciences Centre, Vancouver, BC, Canada) [25, 26]. SOAPdenovo-Trans assembler [27] was also used because the ABySS may misassemble highly expressed transcripts. Assemblies were then joined using BLAST and Cap3 assembler [28]. All coding sequences (CDS) from SG and FB were selected based on similarities with known proteins or containing signal peptide using an automated pipeline [29]. The CDS and their respective protein sequences were placed in a hyperlinked Excel spreadsheet [30]. Software from the Center for Biological Sequence Analysis (Technical University of Denmark, Lyngby, Denmark) were used to predict Signal peptide, transmembrane domains, furin cleavage sites, and mucin-type glycosylation [29, 31–33]. Blastn [34] was used to map the reads into contigs with a word size of 25. The resulting contigs and RPKM values were also mapped to the Excel spreadsheet available as supplemental S1 and S2 Spreadsheets. Differential expression of the reads mapping to contigs between the two libraries were done using the X^2^ test. Relative expression of the transcripts of each separate transcriptome was evaluated using the “expression index”, which is the number of reads to a particular CDS divided by the largest number of reads mapped for a single CDS. The automated annotation of the proteins was based in the matches to various databases, including Gene Ontology, Pfam, Swissprot, KOG, SMART, Refseq-invertebrates and sequences containing Hemiptera[organism] protein sequences obtained from GenBank. The manual annotation was performed as detailed in [28].

### Phylogenetic analysis

Evolutionary analyses were conducted in MEGA6 [35]. The evolutionary history of selected protein sequences was inferred using the Neighbor-Joining method. The percentage of replicate trees in which the associated taxa clustered together in the bootstrap test (1000 replicates) are shown next to the figure branches [36]. The trees were drawn to scale, with branch lengths in the same units as those of the evolutionary distances used to infer the phylogenetic tree. The evolutionary distances were computed using the Poisson correction method [37] and are in the units of the number of amino acid substitutions per site. The rate variation among sites was modeled with a gamma distribution (shape parameter = 1). The sequences are shown with the first 3 letters of the genus name followed by the first 3 of the species name followed by their GenBank accession code.

### qRT-PCR

Tissue expression of randomly chosen sixty genes were evaluated by RT-PCR. Briefly, RNA of salivary glands and fat body were extracted using a RNA SV Total RNA Isolation System (Promega, USA) according to the manufacturer’s recommendations. The cDNA was prepared using a High-Capacity cDNA Reverse Transcription Kit (Applied Biosystems, USA) according to the manufacturer’s recommendations and samples were then frozen at −20°C until analysis. Gene expression was evaluated using a Sybr Green Master Mix (Roche, EUA) and specific primers (forward and reverse) as described in S1 Spreadsheet. Ultrapure DNA/RNA-free water was used as negative control. Relative gene expression was determined by ΔΔCT comparative method using PhSigP-51408_FR4_55–276 as reference gene (similar expression in salivary gland and fat body).

## Results and discussion

### General description of salivary gland and fat body transcriptome

The final assembly of both transcriptomes generated a total of 11,507 CDS, which were mapped from a total of 164,676,091 reads. The 11,507 CDS were subdivided per their putative function (Table 1 and S1 Spreadsheet) as Housekeeping, Secreted, Viral, Transposons and Unknown. The housekeeping (H) class had 5,460 CDS, corresponding to 47% of the total. The putative secreted (S) had 2,943 CDS, or 25% of all CDS. Remarkably, 20% of the reads mapped to transcripts coding for putative viral proteins, particularly to *Triatoma virus* proteins. Transposable elements (TE) accounted to only 3% of the CDS and 0.82% of the reads. Approximately 7% of the reads, corresponding to 2,734 CDS, were not classified and were placed in the unknown (U) class (Table 1).

**Table 1:** General classification of all coding sequences (CDS) from the combined salivary glands and fat body transcriptome of *P*. *lignarius*.

Class	Number of CDS	% of total	Number of Reads	% of total
**Housekeeping**	5,460	47.45	80,227,208	48.72
**Secreted**	2,943	25.58	38,247,116	23.23
**Viral**	12	0.10	33,602,364	20.41
**Transposons**	358	3.11	1,343,408	0.82
**Unknown**	2,734	23.76	11,255,995	6.84
**Total**	11,507	100	164,676,091	100

### Housekeeping (H) genes of the combined transcriptome

The 5,460 CDS classified as housekeeping genes were characterized in 21 subgroups depending of their putative functions (S1 Spreadsheet and Table 2). In these subgroups, the category “signal transduction” had the highest expression presenting 11% of the mapped reads from class H, followed by “storage” and “protein synthesis machinery”.

**Table 2:** Classification of all coding sequences (CDS) with putative housekeeping function extracted from the transcriptome of *P*.*lignarius*.

Class	Number of Contigs	% of total	Number of Reads	% of total
**Signal transduction**	1,009	18.48	9,118,215	11.37
**Storage**	18	0.33	7,216,235	8.99
**Protein synthesis machinery**	280	5.13	6,379,967	7.95
**Nuclear regulation**	315	5.77	6,211,075	7.74
**Transcription machinery**	595	10.90	6,207,164	7.74
**Transporters/storage**	458	8.39	5,503,602	6.86
**Protein modification machinery**	311	5.70	4,996,742	6.23
**Cytoskeletal**	329	6.03	4,788,334	5.97
**Metabolism, lipid**	282	5.16	4,584,580	5.71
**Extracellular matrix/cell adhesion**	199	3.64	3,512,502	4.38
**Proteasome machinery**	242	4.43	3,112,153	3.88
**Metabolism, energy**	190	3.48	3,011,966	3.75
**Metabolism, amino acid**	116	2.12	2,696,206	3.36
**Metabolism, carbohydrate**	191	3.50	2,537,516	3.16
**Oxidant metabolism/detoxification**	144	2.64	2,378,367	2.96
**Protein export machinery**	331	6.06	2,360,314	2.94
**Immunity**	105	1.92	2,124,090	2.65
**Metabolism, nucleotide**	103	1.89	1,199,974	1.50
**Metabolism, intermediate**	60	1.10	978,596	1.22
**Transcription factor**	148	2.71	946,838	1.18
**Nuclear export**	34	0.62	362,772	0.45
**Total**	5,460	100	80,227,208	100

**Fig 1:**
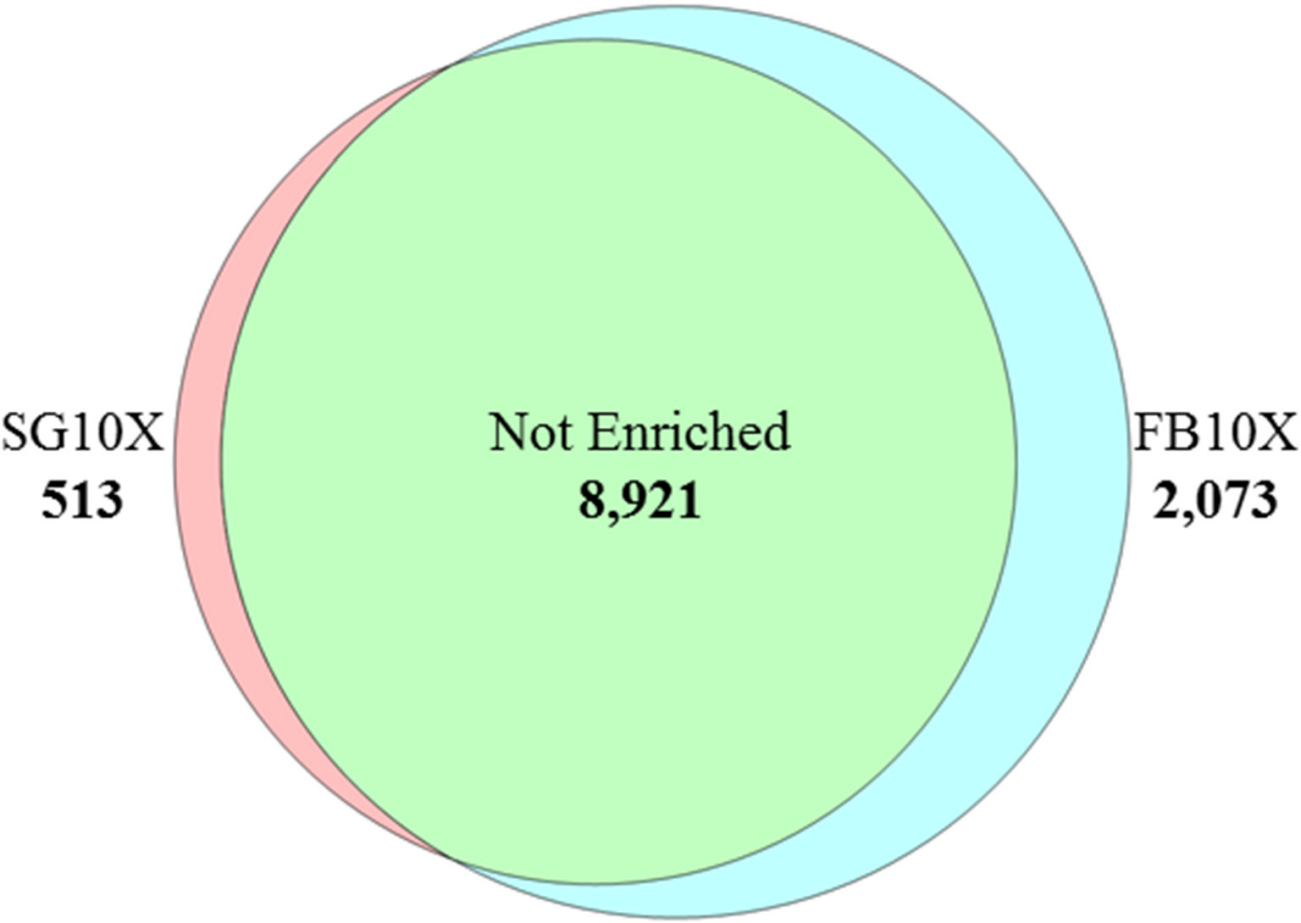
Venn diagram of SG and FB transcriptomes of *P*. *lignarius*. SG10x, 10-fold overexpressed transcripts in the salivary gland compared to fat body; FB10x, 10-fold overexpressed transcripts in the fat body compared to the salivary gland; and, Not Enriched, transcripts expressed in both SG and FB with less than a 10-fold variation.

Of the 11,507 CDS of the combined transcriptome, 513 were found 10x or more expressed in the salivary glands, 2,073 were found similarly overexpressed in the fat bodies, and 8,921 CDS were not particularly overexpressed in either organ (Fig 1). We will proceed analyzing these enriched subsets, as they represent what is possibly specific for each tissue type.

### The enriched salivary gland transcriptome of *Panstrongylus lignarius*

A total of 513 CDS appeared at least 10 X overexpressed in salivary glands (now referred to SG enriched transcriptome) when compared to the fat body transcriptome (S2 Spreadsheet and Table 3). The majority of these were associated with secreted products as expected for the SG.

**Table 3:** Classification of coding sequences (CDS) that are at least 10 x overexpressed in the salivary glands transcriptome as compared to the fat body transcriptome.

SG overexpressed 10 x	Average FPKM SG	N	% of total	Average FPKM FB	Ratio FPKM
**Secreted**	2,005.45	328	64.13	6.66	301.07
**Housekeeping**	221.60	136	26.51	4.28	51.74
**Unknown**	830.20	44	8.58	3.95	210.38
**Transposons**	6.37	4	0.78	0.31	20.58
					
**Total or average**		513	100		145.94

The SG enriched transcriptome contains 136 transcripts attributed to the housekeeping class, as further detailed in Table 4.

**Table 4:** Classification of coding sequences (CDS) 10X overexpressed in the salivary transcriptome with putative housekeeping function.

Subclass	No. of CDS	No. of reads	% Total
Detoxification	17	673,883	34.84
Cytoskeletal	4	267,216	13.84
Lipid metabolism	18	203,530	10.54
Protein synthesis machinery	4	185,847	9.62
Unknown conserved	15	143,472	7.42
Transporters	33	140,626	7.28
Signal transduction	24	89,506	4.63
Glycosyl transferase	1	76,195	3.95
15-hydroxyprostaglandin dehydrogenase	4	65,330	3.38
Transcription machinery	6	52,714	2 2.72
Nuclear Regulation	2	23,056	1.19
Salivary ubiquitin	1	3,581	0.18
Transcription Factors	3	2,156	0.11
Protein export and modification	2	2,114	0.11
Intermediate metabolism	1	1,181	0.06
Energy metabolism	1	644	0.03
**Total**	**136**	**1,931,051**	**100**

Transcripts belonging to the detoxification class are the most abundant of the SG overexpressed CDS of the housekeeping class. Of these 17 transcripts, 12 are members of the cytochrome P450 family. Additionally, 4 CDS matches 15-hydroxyprostaglandin dehydrogenase, a similar finding in previous triatomine transcriptomes of *Triatoma* [18, 38, 39] and *Panstrongylus megistus* [40]. In a previous review, it was stated that this combination of transcripts suggested a role of triatomine salivary glands in the manufacture of eicosanoids [9]. However, a search for prostaglandins in the saliva of triatomines was negative [16]. Since these enzymes are associated with prostaglandin catabolism, it is here speculated that prostaglandins may function as salivary secretagogues and that the enzyme is associated with agonist detoxification. Alternatively, non-prostaglandin eicosanoids may be produced by the SG.

### Transcripts coding for putative secreted proteins in *Panstrongylus lignarius* salivary glands

Lipocalins and the small molecule binding proteins with a JH binding motif comprised over 40% of the secreted transcripts that are overexpressed in the SG transcriptome (Table 5).

**Table 5:** Classification of coding sequences with putative secretory function extracted from sialotranscriptome of *P*. *lignarius*.

Subclass	No. of CDS	No. of reads	% Total
**Lipocalins**	88	6,280,411	29.09
**JH binding protein**	7	2,629,387	12.18
**Kazal-type peptides**	18	2,343,412	10.85
**Glycine rich proteins**	17	1,951,574	9.04
**Salivary proteases**	33	1,829,373	8.47
**Other secreted proteins**	94	1,580,689	7.32
**Conserved insect family 15**	4	1,349,810	6.25
**Conserved insect family 12**	17	1,000,854	4.64
**Immunity related**	8	983,020	4.55
**Inositol-145-triphosphate 5-phosphatase**	4	414,855	1.92
**Amylase/Maltase**	4	302,186	1.40
**Antigen-5**	3	245,873	1.14
**Hemiptera specific family 225**	5	171,874	0.80
**Mucin**	3	155,075	0.72
**Serpin**	2	86,723	0.40
**Apyrase**	2	83,292	0.39
**Hemiptera specific family 210**	10	51,196	0.24
**Transferrin**	1	35,869	0.17
**Endonuclease**	1	35,862	0.17
**Ribonuclease**	1	28,532	0.13
**Lipase**	3	12,443	0.06
**Phosphatase**	1	11,308	0.05
**Odorant binding protein**	2	5,412	0.03
Total	328	21,589,030	100.00

Lipocalins are widely distributed in vertebrates, invertebrates, plants and bacteria [41, 42] and it is one of the main classes of proteins on the salivary glands of ticks and triatomines [43, 44]. Lipocalins possess a conserved three-dimensional structure and include an extensive group of extracellular proteins that generally bind to small hydrophobic proteins, extracellular ligands and other proteins. Triatomine lipocalins were shown to have vasodilator, anticoagulant and antiplatelet activities [45]. Some of these functions, such as the anticlotting activities of triabin [46] or nitrophorin 2 [47, 48], are exerted by interactions of the lipocalin with a clotting cascade protein, while other functions relate to their strong binding to agonists of hemostasis or inflammation, namely their kratagonist function (from the Greek “kratos” = to seize) [49]. Recently, a salivary lipocalin of *Rhodnius prolixus* was shown to antagonize cysteinyl leukotrienes [50].

**Fig 2:**
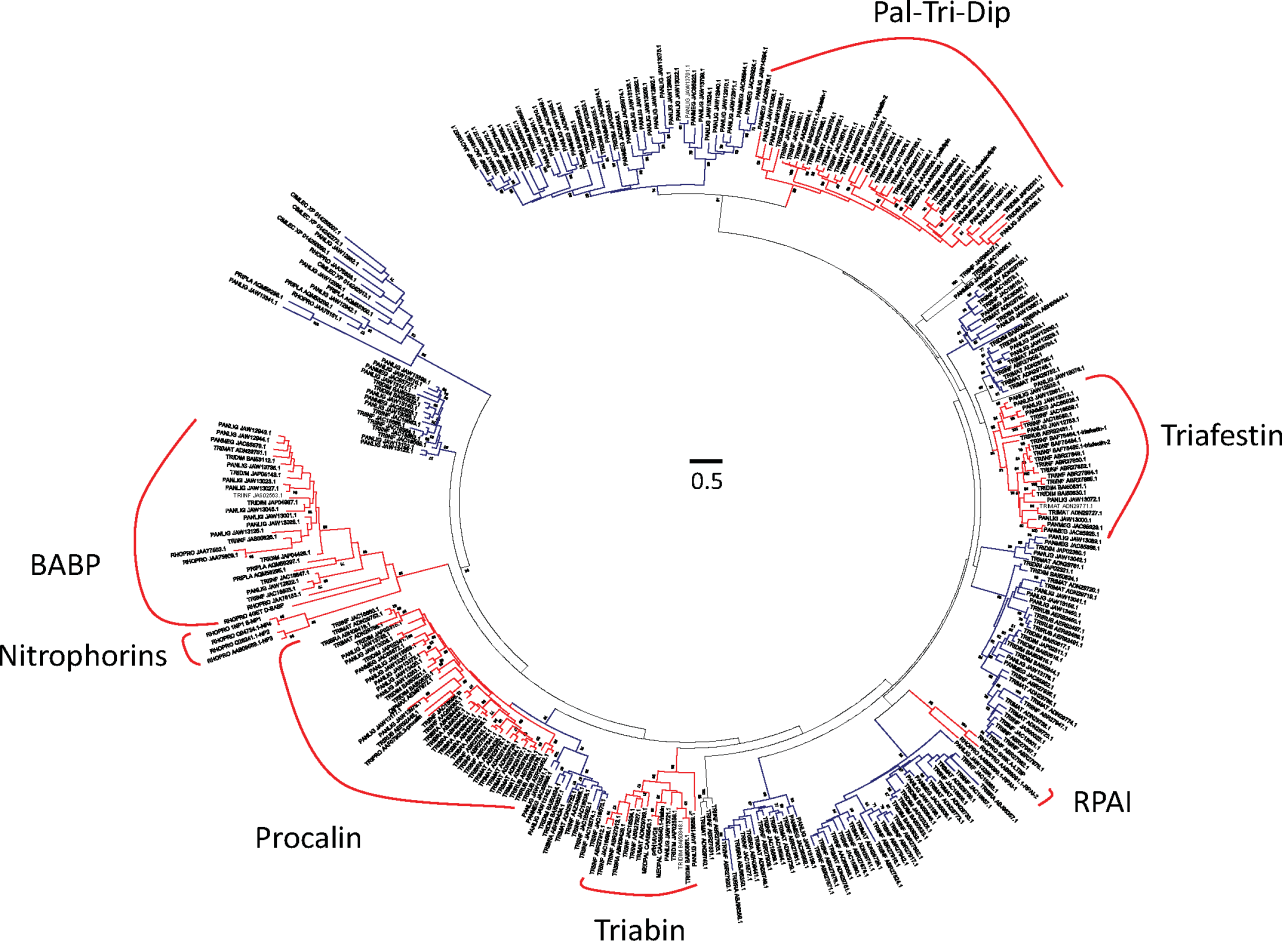
Phylogram of the lipocalin family proteins from *P*. *lignarius* and their best matches. The optimal tree with the sum of branch length = 123.81570372 is shown. The tree is drawn to scale, with branch lengths in the same units as those of the evolutionary distances used to infer the phylogenetic tree. The bar at the center of the graph indicates a value of 0.5. The analysis involved 330 amino acid sequences. All ambiguous positions were removed for each sequence pair. There were a total of 423 positions in the final dataset. The values near the branches represent the percentage of bootstrap support. Values below 50% are not shown. For more details, see Material and Methods.

The assembly of the *P*. *lignarius* transcriptome revealed 78 contigs coding for full length lipocalins, all at least 10 times overexpressed in the SG when compared to the FB, and averaging over 3,000 fold overexpression. The protein sequences of these 78 gene products were aligned with 252 other triatomine lipocalins, producing a dendrogram where at least 15 clades with strong bootstrap support are observed (Fig 2). Eight *P*. *lignarius* sequences are found within the Pal-Tri-Dip clade which includes the platelet aggregation inhibitors pallidipin [51, 52], triplatin [53, 54] and dipetalodipin [55] from the *Triatoma* and *Dipetalogaster* genera. Triplatin and dipetalodipin were shown to be kratagonists of eicosanoids, possibly the same mechanism occurring in pallidipin. Triafestins are inhibitors of the activation of the kinin system [56] found in *T*. *infestans*. The two characterized sequences are within a clade with strong bootstrap support. Four *P*. *lignarius* sequences are found within this clade. The clade containing *Rhodnius* platelet aggregation inhibitor (RPAI) [57, 58], as well the *Rhodnius prolixus* leukotriene binding protein (LBP) [50] is *Rhodnius* specific, thus not containing any *Panstrongylus* sequences. It, however, has a sister clade, with low bootstrap support, containing several *Triatoma* and *Panstrongylus* sequences, including two from *P*. *lignarius*. The clade named triabin contains the anti-thrombin inhibitor from *T*. *infestans* [46, 59] and several other sequences, including two from *P*. *lignarius*. A large clade contains the salivary antigen procalin [60], of unknown physiological function. The BABP clade, which is sister to the uniquely *Rhodnius* nitrophorin clade, has the *Rhodnius prolixus* Biogenic Amine Binding Protein [61, 62], a protein having anti-platelet and vasodilatory activities. Ten *P*. *lignarius* sequences populate this clade. This analysis may help to design experiments with recombinant triatomine proteins aiming at determining their functions.

**Fig 3:**
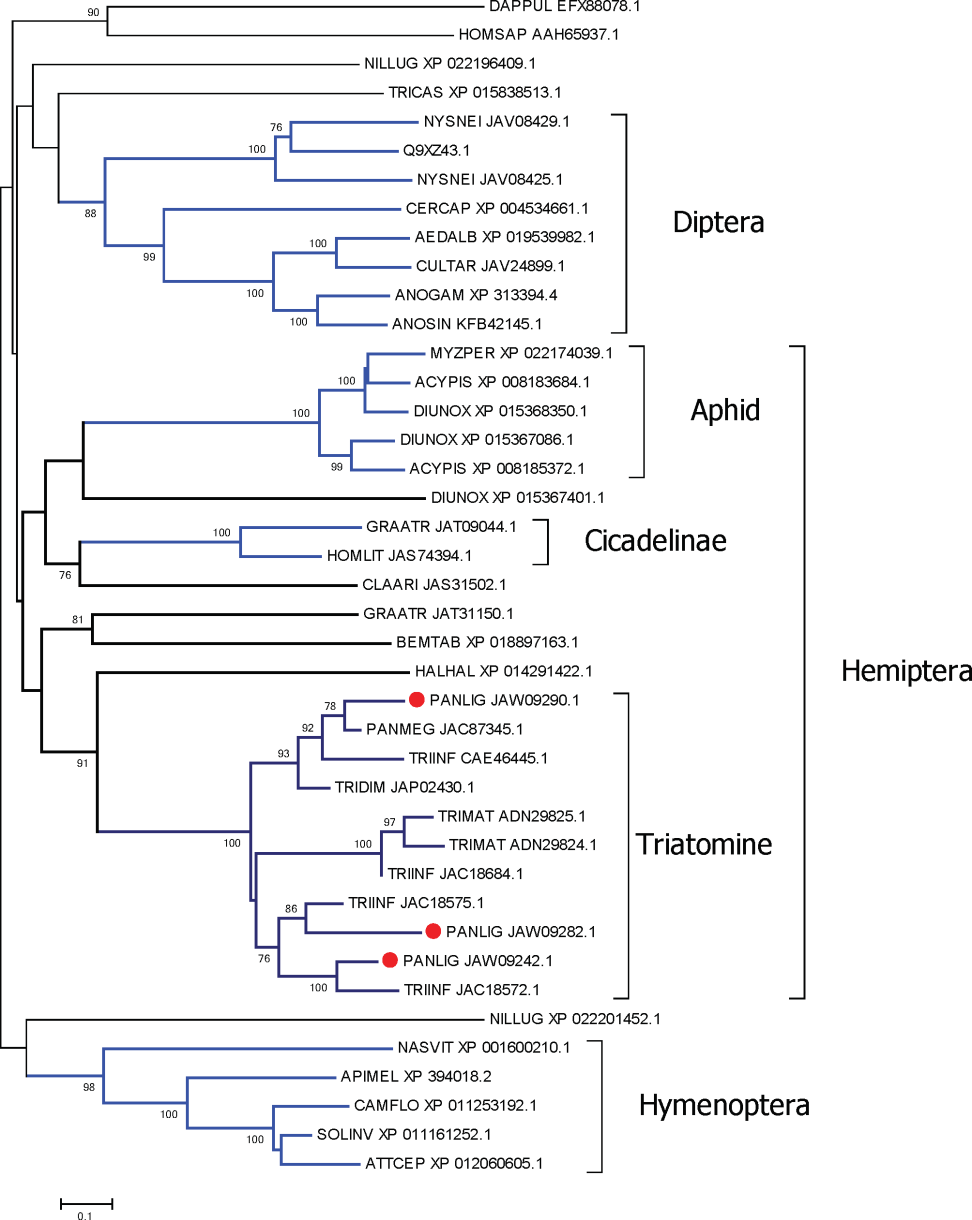
Phylogram of the apyrase/5’-nucleotidase family of proteins from *P*. *lignarius* and their best matches. The optimal tree with the sum of branch length = 15.40595903 is shown. The values near the branches represent the percentage of bootstrap support. Values below 75% are not shown. The analysis involved 41 amino acid sequences. All ambiguous positions were removed for each sequence pair. There were a total of 689 positions in the final dataset. For more details, see Material and Methods.

Apyrases are enzymes that hydrolyze ATP and ADP to AMP and orthophosphate. These are abundant in the saliva of blood feeding arthropods presumably because they destroy these agonists of platelet aggregation and inflammation [63]. In mosquitoes [64] and triatomine bugs [65], but not in *Rhodnius*, salivary apyrases belong to the 5’-nucleotidase family, while in *Cimex* [66] and sand flies [67], and probably in *Rhodnius* [68], they belong to the *Cimex* family of apyrases. In *P*. *lignarius*, two apyrase-like proteins of the 5’-nucleotidase family are highly expressed in the SG with a total of 83,000 reads (Table 5). A third member of the family was additionally identified, but it is not particularly enriched in either FB or SG transcriptomes. This third member has a glycophosphatidylinositol (GPI) anchor as predicted by the big-PI Predictor site [69], while the two overexpressed proteins do not, indicating they are secreted and not membrane bound. This is in accordance with the postulated evolution of secreted salivary apyrases which included a step of gene duplication of an ancestral, membrane-bound product, plus loss of the GPI anchor [64]. The phylogram of the three *P*. *lignarius* members of the apyrase/5’-nucleotidase family together with their best matches by Blastp to GenBank proteins displays robust clades for various insect orders of families, the *P*. *lignarius* proteins each sharing a robust clade with other triatomine proteins, indicative of their long evolutionary history (Fig 3). Tellingly is the absence of *Rhodnius* proteins, supporting the monophyletic status of this genus with relation to its evolution to blood feeding [70].

The inositol phosphate 5-phosphatase (IPP) enzymes were predicted in 4 CDS. Previously, the *Rhodnius* homolog was shown to act both on soluble inositol phosphatase and the substrate phosphoinositide [71]. These phospholipids are involved in several cellular processes related to signal transduction, secretion and cytoskeletal structure. Although IPP was found to be produced by *Rhodnius prolixus* salivary glands in 2006 [71], it still has an unknown function in this organism. These studies suggested that IPP ejected in saliva act by decreasing the concentration of PI (4,5) P2 e PI(3,4,5) P3, which are present in the plasma membrane of cells and platelets, causing changes in the cytoskeletal architecture [72]; however, how they could enter the cell to perform this function is still a puzzle.

Among other enzymes overexpressed in the salivary glands, we address a cathepsin D, which is normally a housekeeping enzyme associated with lysosomes, but is over 1,000 fold overexpressed in the SG, and has a signal peptide indicative of secretion, indicating this protein may have a role in blood meal acquisition by interfering with host hemostasis. Serine proteases were also found, these having been regularly detected in triatomine sialomes [9]. The function of these proteases in feeding, however, is unknown, but could be related to fibrin hydrolysis, as occur with salivary serine proteases from a tabanid [73].

Somewhat surprising is the finding of amylases and maltase overexpressed in the SG. These enzymes are usually found in mosquito sialomes, and are associated with the sugar feeding mode of these organism [63]. Is it possible that *P*. *lignarius* feeds on plants? Recently it was shown that *R*. *prolixus* feeds on plants [74] and perhaps this behavior is more widespread.

**Fig 4:**
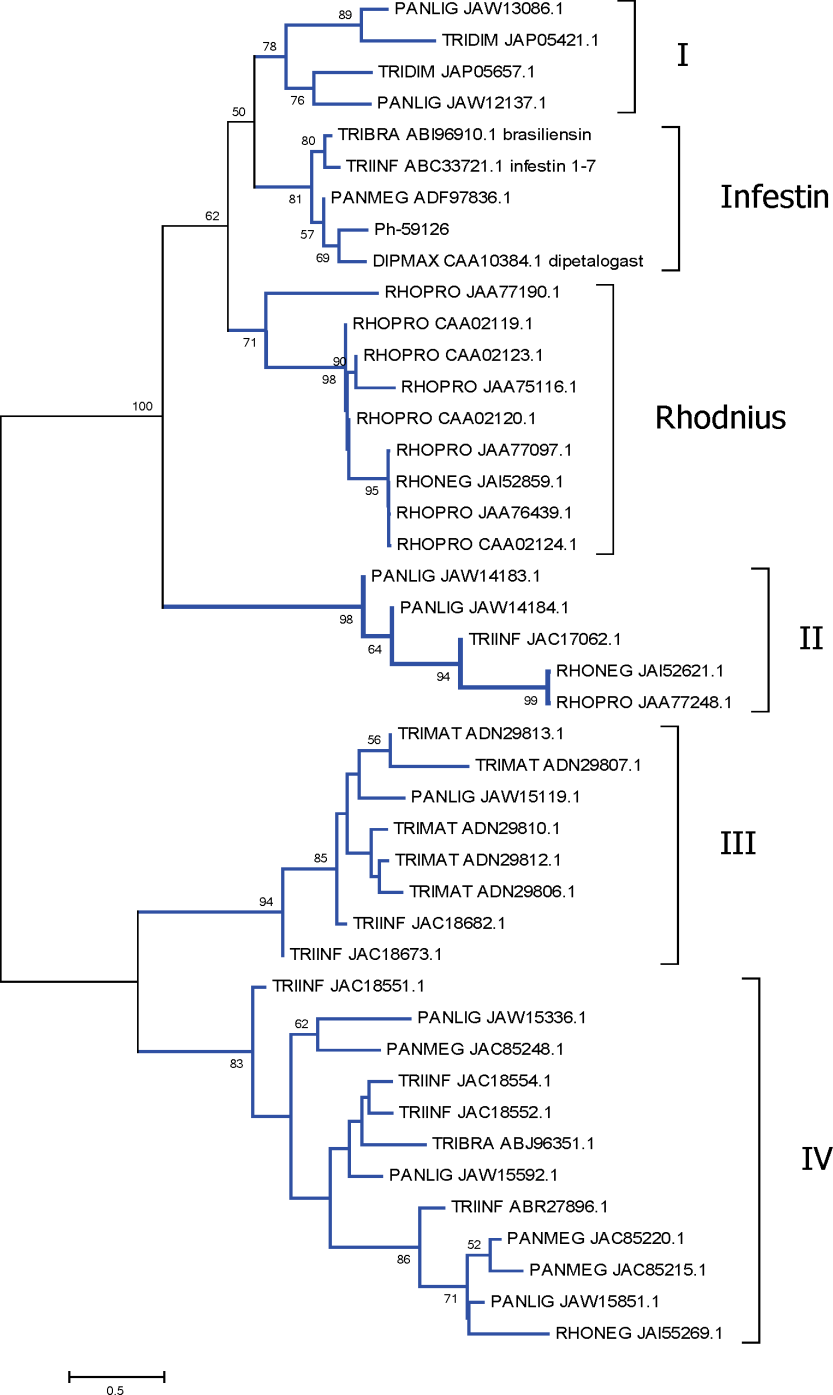
Phylogram of the Kazal-domain containing family of proteins from *P*. *lignarius* and their best matches. The optimal tree with the sum of branch length = 14.99541793 is shown. The values near the branches represent the percentage of bootstrap support. Values below 50% are not shown. The analysis involved 43 amino acid sequences. All ambiguous positions were removed for each sequence pair. There were a total of 969 positions in the final dataset. For more details, see Material and Methods. Robust clades are named I-IV (where no member has been functionally characterized); Infestin and Rhodnius indicate clades containing previously functionally characterized proteins.

Serine-proteases inhibitory proteins were found in the salivary gland transcriptome. These may play a role in inhibiting the coagulation cascade or the activation of the complement system. These proteins are subdivided according to their domain, such as the Kazal domain and serpins [75]. The Kazal family was the second most abundant in *P*. *lignarius* SG, corresponding to ~ 10% of the total reads. They consist in molecules with single or multiple domains with a shared conserved motif and a distinct pattern of cysteine distribution. Several proteins from the Kazal family were already described in vertebrates and invertebrates, including triatomines. Kazal domain containing peptides are typical inhibitors of serine proteases. Indeed, the two Kazal domain protein Rhodnin, isolated from the crop of *R*. *prolixus*, was shown to inhibit thrombin [76]; similarly, dipetalogastin was isolated from *D*. *maximus* [77] and brasiliensin from *T*. *brasiliensis* [78] guts. Infestins, with up to seven Kazal domains, were isolated from *T*. *infestan*s midgut [79–82] and shown to inhibit thrombin, neutrophil elastase and Factor XIIa. The salivary gland transcriptome of *P*. *lignarius* discloses a seven-Kazal domain containing peptide that is the most expressed member of this peptide class, encoded by Ph-59126. On the other hand, a KGD-containing Kazal peptide in tabanids named vasotab was shown to have vasodilatory activity, in addition to anti-platelet activity [83]. Phylogenetic analysis of *P*. *lignarius* Kazal-domain containing peptides aligned with other related triatomine proteins indicates the complexity of this family (Fig 4). A clade containing the triatomine intestinal serine protease inhibitors (named Infestin in Fig 4) contains the seven-Kazal peptide from *P*. *lignarius* mentioned above, indicating that *P*. *lignarius* may have co-opted this peptide family for salivary expression. Four other robust clades, plus one *Rhodnius*-specific clade indicate the diversity of these peptides in triatomines. Clade IV includes JAW15592.1, JAW15851.1 and JAW15336.1 which have weak similarity to vasotab; however, the KGD domain of vasotab associated with anti-platelet function is not found in *P*. *lignarius*.

The serpin domain containing proteins, or serpin-like, are also found in the saliva of arthropods and they affect hemostasis, including platelet adhesion, fibrinolysis and coagulation, facilitating the blood feeding. Two salivary enriched serpins were found in the *P*. *lignarius* transcriptome.

The Antigen-5 protein family is a group of proteins belonging to the cysteine-rich secretory proteins (CRISP) superfamily which have been identified through sialotranscriptomes in the saliva of different hematophagous insects, such as mosquitoes [84, 85], phlebotomines [86–88], and triatomines [17, 18, 21, 22, 24, 89–91]. Although it is frequently found in the saliva of hematophagous arthropods, its function is mostly unknown and it was described to be part of the toxin repertoire of snake venom [92]. An antigen-5 protein from *Dipetalogaster maxima* was shown to inhibit platelet aggregation by low doses of collagen and to have superoxide dismutase activity [93]. The current transcriptome of *P*. *lignarius* identified three members of this family that are highly expressed in the SG.

Immunity-related proteins and peptides are broadly found in several organisms from mammals to plants and hematophagous arthropods. In the saliva of these insects and ticks, those peptides, such as lysozyme and defensins may help in controlling the microbial growth in the ingested blood and, in the vertebrate host, they may prevent microbial infections at the biting place [94]. Pathogen pattern recognition proteins such as lectins were also found in this group; these proteins may play a role in modulating host immunity.

Only two transcripts coding for members of the Odorant/Pheromone-Binding Family (OBP) were found in the enriched SG transcriptome, corroborating the findings from other studies with *Rhodnius*, *Triatoma*, *Panstrongylus* and *Cimex*, where in *Cimex* they were more abundant. The properties of these proteins in blood-feeding is unknown [9]. We also identified 7 CDS putatively coding for Juvenile hormone binding proteins (JH binding proteins) with very long sequences and representing ~12% of the total reads. These proteins were already identified in the SG of *Rhodnius neglectus* [91], *Anopheles culicifacies* [95], *Anopheles gambiae* [96] and *Aedes aegypti* [97], however, no single CDS coding for JH binding proteins was found in the sialotranscriptome of *P*. *megistus* [24]. JH in adult insects is responsible for controlling the reproductive maturation and inducing vitellogenesis, as JH and vitellogenin are negatively correlated. Additionally, JH carrier and odorant binding proteins have a pivotal function in the regulation of feeding behavior of hematophagous arthropods [98]. Despite these more conventional functions, it is possible that these gene products may have been recruited to a salivary function to act as kratagonists of lipidic mediators of hemostasis.

The sialome of *P*. *lignarius* revealed at least two protein family expansions that appear exclusive of Hemiptera, and three protein family expansions that appear to be exclusive of insects. Some of the transcripts are highly expressed, such as Ph-55919, with a RPKM = 14,784. Their functions are unknown.

### Fat body

There are few transcripts analyses of fat body published so far, such as *Bactocera dorsalis* [12], *Melipona scutellaris* [99], and *Aedes aegypti* [100], and none are related to triatomines.

The evaluation of the entire transcriptome from the fat body of *P*. *lignarius* (Table 6) showed the most prevalent subclass was classified as secreted proteins with ~20.5% of the total reads, which agrees with one of the main functions of this tissue, the synthesis of peptides with distinct functions, many to be destined to the hemolymph compartment. Following the secreted proteins, the next subclass with high FB expression was storage with ~8% of the reads, also associated with secreted hemolymph proteins. On the 10x overexpressed subset (Table 7), the most prevalent classes of molecules were also related to secreted proteins and storage, followed by proteins with unknown function and cytoskeletal proteins.

**Table 6:** Classification of all coding sequences (CDS) from the fat body transcriptome of *P*. *lignarius*.

Subclass	No. of CDS	No. of reads	% Total
Secreted protein	2,664	16,900,616	20.40
Storage	17	6,882,176	8.23
Signal transduction	985	5,648,555	6.82
Transcription machinery	590	4,808,076	5.80
Viral product	11	4,200,886	5.07
Protein synthesis machinery	276	4,173,641	5.04
Transporters and channels	425	3,879,540	4.68
Unknown product	1,559	3,429,273	4.14
Cytoskeletal proteins	325	3,399,663	4.10
Unknown conserved	881	3,356,864	4.05
Nuclear regulation	313	2,975,729	3.60
Protein modification	295	2,844,094	3.43
Proteasome machinery	241	2,378,803	2.87
Lipid metabolism	260	2,260,869	2.73
Amino acid metabolism	115	2,250,741	2.71
Energy metabolism	188	2,075,093	2.50
Carbohydrate metabolism	185	1,698,819	2.05
Protein export	329	1,392,878	1.68
Extracellular matrix	182	1,298,620	1.56
Unkown conserved membrane protein	235	1,029,230	1.24
Transposable element	353	1,020,635	1.23
Nucleotide metabolism	102	979,483	1.18
Immunity	97	922,667	1.11
Intermediary metabolism	59	789,404	0.95
Detoxification	72	762,671	0.92
Transcription factor	145	677,034	0.81
Oxidant metabolism/Detoxification	56	496,585	0.60
Nuclear export	34	287,566	0.34
**Total**	**10,994**	**82,820,211**	**100**

**Table 7:** Coding sequences (CDS) 10X overexpressed in fat body compared to salivary glands from transcriptome of *P*. *lignarius*.

Subclass	No. of CDS	No. of reads	% Total
Secreted protein	611	11,982,880	32.26
Storage	9	6,803,966	18.32
Unknown product	387	2,081,905	5.60
Cytoskeletal proteins	112	1,794,794	4.83
Transcription machinery	45	1,696,142	4.57
Transporters and channels	89	1,609,127	4.33
Signal Transduction	168	1,242,131	3.34
Amino acid metabolism	35	1,065,122	2.87
Unknown conserved	156	1,000,574	2.69
Carbohydrate metabolism	30	826,799	2.23
Energy metabolism	33	799,257	2.15
Proteasome machinery	29	717,203	1.93
Extracellular matrix	52	681,489	1.83
Nucleotide metabolism	11	677,461	1.82
Protein modification	65	561,469	1.51
Transposable element	18	494,232	1.33
Detoxification	9	490,735	1.32
Unkown conserved membrane protein	31	459,387	1.24
Lipid metabolism	43	450,227	1.21
Protein synthesis machinery	16	390,148	1.05
Immunity	31	346,241	0.93
Nuclear regulation	22	283,827	0.76
Intermediary metabolism	6	230,441	0.62
Oxidant metabolism/Detoxification	14	195,373	0.53
Protein export	32	136,685	0.37
Transcription factor	17	73,442	0.20
Nuclear export	2	53,368	0.14
**Total**	**2,073**	**37,144,425**	**100**

**Fig 5:**
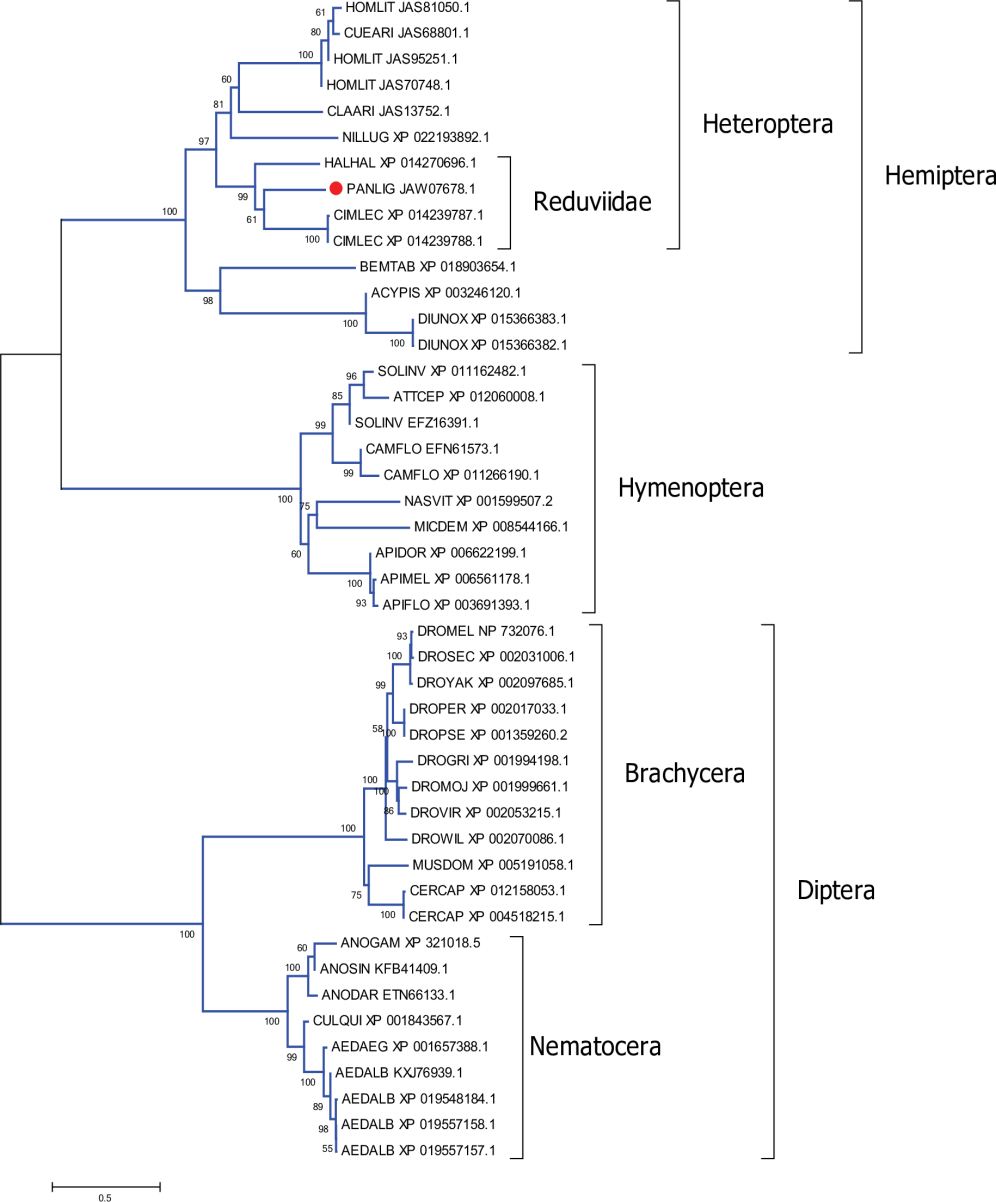
Phylogram of the vitellogenin protein of *P*. *lignarius* and their best matches. The optimal tree with the sum of branch length = 11.41384103 is shown. The values near the branches represent the percentage of bootstrap support. Values below 50% are not shown. The analysis involved 45 amino acid sequences. All ambiguous positions were removed for each sequence pair. There were a total of 1713 positions in the final dataset. For more details, see Material and Methods. The *Panstrongylus lignarius* sequence is shown with a red marker.

Vitellogenin is an important protein precursor of vitelline, used in insect oocytes’ formation and maturation, and it is produced exclusively in the fat body of insects and then processed and secreted in hemolymph [101]. The transcriptome of *P*. *lignarius* allowed for the disclosure of JAW07678.1, a 1,324 aa long protein with N-terminal Vitellogenin-N and carboxyterminal VWD motifs, typical of insect vitellogenins. Alignment of this protein sequence with its best matches from the NCBI database allows identification of the insect order-specific clades Hemiptera, Hymenoptera and Diptera, including the Diptera sub-orders Brachycera and Nematocera, and the Heteroptera sub-order within the Hemiptera, and within Heteroptera, the Reduvidae family (Fig 5).

**Fig 6:**
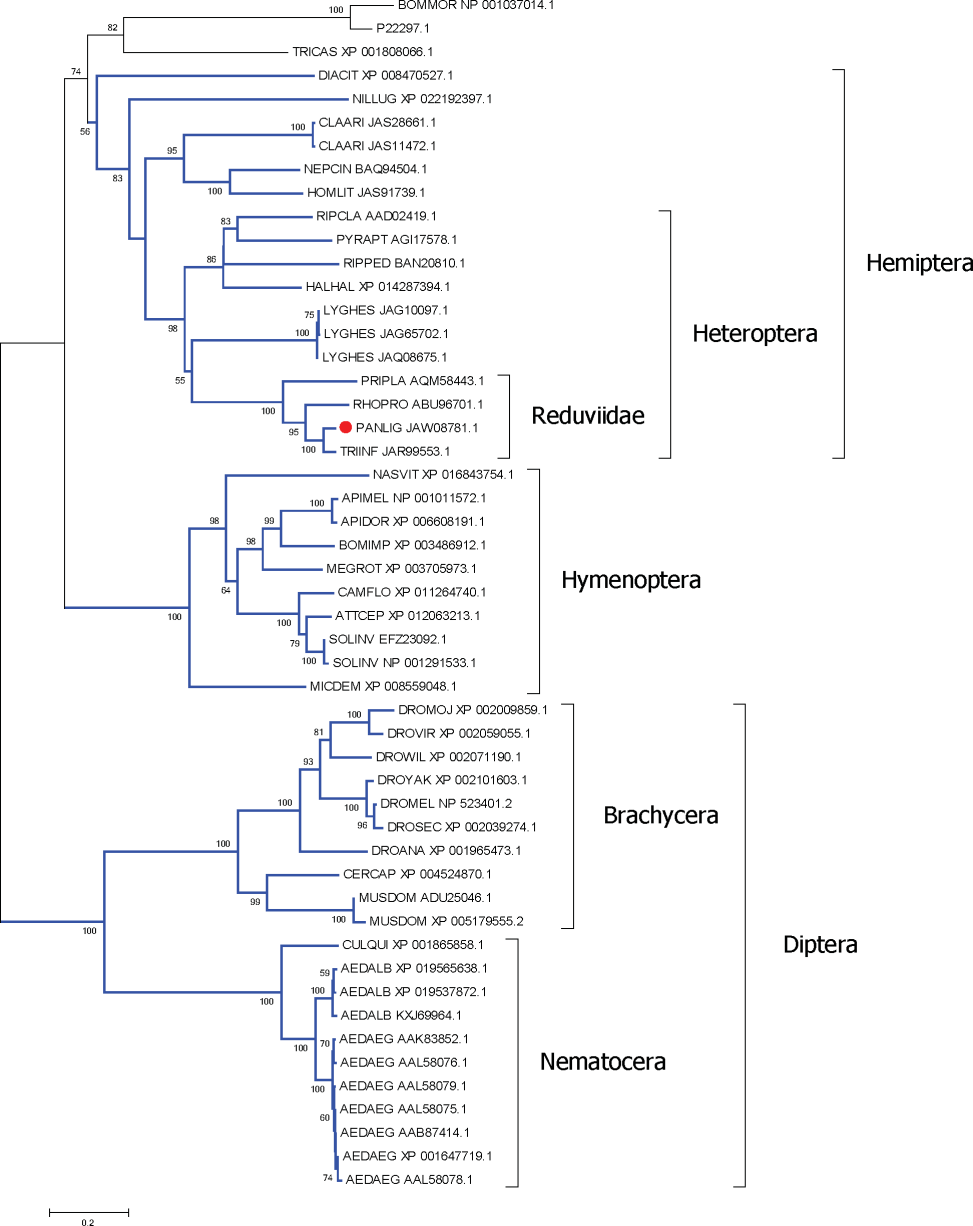
Phylogram of the transferrin protein of *P*. *lignarius* and their best matches. The optimal tree with the sum of branch length = 10.61177438 is shown. The values near the branches represent the percentage of bootstrap support. Values below 50% are not shown. The analysis involved 51 amino acid sequences. All ambiguous positions were removed for each sequence pair. There were a total of 1487 positions in the final dataset. For more details, see Material and Methods. The *Panstrongylus lignarius* sequence is shown with a red marker.

Transferrins are glycoproteins found in different animals, such as mammals, marsupials, fish and in more than 34 species of invertebrates, including *R*. *prolixus* [102]. These proteins may function in insect defense mechanisms [103]. In insects, transferrins are synthesized and stored in the fat body for posterior secretion to the hemolymph, where they participate in iron uptake and distribution with ferritin [104]. A transferrin was identified in the fat body transcriptome of *P*. *lignarius*. This 656 aa long protein was aligned with its best matches from the NCBI database to produce the phylogram shown in Fig 6, where robust clades of several insect orders are observed.

### Triatoma virus

Previous sialotranscriptomes from triatomines reported a low expression of diverse viruses. For example, in an Illumina-based sialotranscriptome of *Panstrongylus megistus* seven transcripts were reported that best matched viral proteins. However, these transcripts were poorly expressed, reaching expression indexes below 0.0025 [40]. Another Illumina-based sialotranscriptome of *Triatoma* infestans reported 38 transcripts similarly coding for putative viruses, two of which had an expression index between 1 and 2.7, most similar to Deformed winged virus and to Drosophila A virus [39]. Similarly, the *P*. *lignarius* transcriptome uncovered 12 transcripts putatively coded by viruses. Remarkably, two transcripts coding for the capsid P1 polyprotein (Genbank AHB63946.1) and the nonstructural protein precursor (Genbank NP_620562.1) of *Triatoma virus* were very highly expressed, attaining expression indexes of 100 (most expressed transcript) and 18 in the SG. Each transcript accrued more than 9% of the totality of reads, summing up to near 20% of all transcriptome reads deriving from the viral genome (Table 1 and S1 Spreadsheet). The relative expression was high both on the salivary gland as well as in the fat body transcriptomes. *Triatoma virus* was first discovered infecting *T*. *infestans* in Argentina [105], and later shown to infect other *Triatoma* species as well as *Psammolestes coreodes* [106]. A survey of laboratory reared insects additionally detected several species harboring the virus, including specimens from the Barbacena insectary from where the *P*. *lignarius* used in this work derived [107]. However, *P*. *lignarius* was not analyzed in that study, and thus this species can be added to the 15 previously found infected with this virus, which included a single *Panstrongylus* species (*P*. *guentheri*).

Mice inoculation with the virus resulted in a non-infective immune response [108], and it was found that people with Chagas’ disease living in Bolivia, Argentina and Mexico developed a detectable immune response to the virus [109]. The high levels of transcription found in the salivary glands suggest that in addition to the previously proposed route of fecal contamination, humans and rodents could be infected via direct salivary inoculation. From the standpoint of insect to insect propagation, it has been proposed that viral transmission occurs via the fecal-oral route or by cleptohematophagy [107]. It should be added that, to the extent mature viral particles are secreted in the bugs’ saliva, co-feeding bugs could promote transmission between insects, as their feeding mechanism includes frequent reversal of the ingestion pump, possibly to dislodge incipient platelet plugs [110, 111], thus spreading the virus in the skin vasculature from where it could reach co-feeding insects.

### Determination of relative tissue expression of randomly selected genes

**Fig 7:**
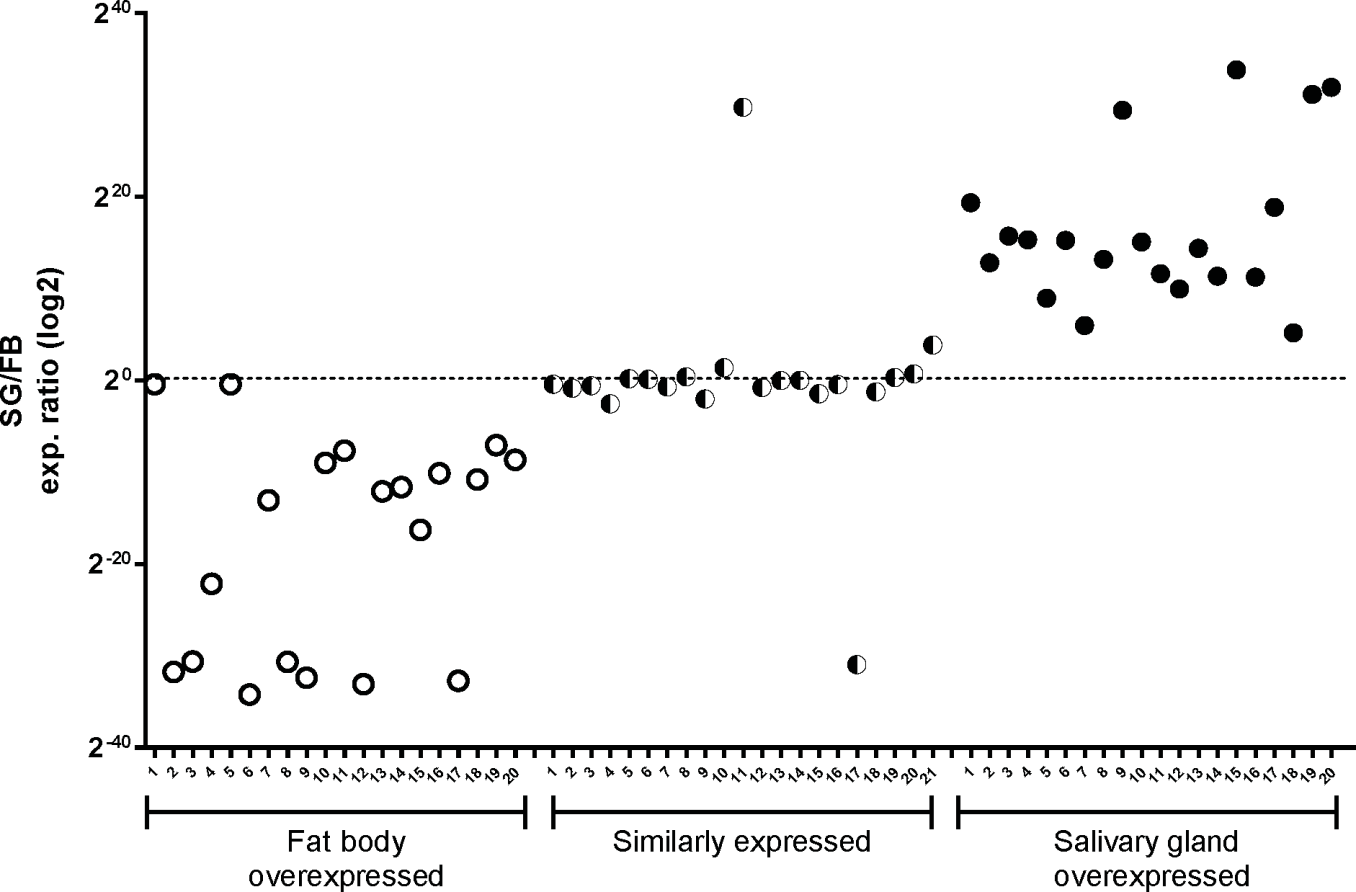
Validation by qPCR of the differential expression of transcripts between salivary gland and fat body libraries. Relative gene expression was calculated by ΔΔCT method using PhSigP-51408_FR4_55–276 as reference gene (similar expression in salivary gland and fat body). Scatter plot presenting the SG:FB expression ratio of FB overexpressed, similarly expressed and SG overexpressed genes. The Y axis is the log2 of the observed ratio between SG and FB by qPCR on three groups of arbitrarily selected transcripts that are overexpressed in either tissue, or similarly expressed. S1 Table provides for the transcript names and primer sequences used in this experiment.

Confirmation of the gene expression of randomly selected genes from the salivary gland and fat body transcriptomes of *P*. *lignarius* was performed. We selected twenty 10X overexpressed SG genes, twenty 10X overexpressed FB genes and twenty genes with similar expression in both tissues. As demonstrated in Fig 7, SG or FB overexpression or similar expression between these tissues was confirmed by qRT-PCR for most of the evaluated genes, as predicted by transcriptome analysis.

### Conclusions

Different species of insects diverge in relation to the molecular compounds of their saliva, which are determined by their evolutionary history including habitat distribution and food source. Sialome studies of several species have already identified a variety of molecules with potential industrial and/or clinical use for their pharmacological activity and discriminating properties as biomarkers of vector exposure, respectively [112]. The present work contributed to the public disclosure of over 9,000 protein sequences that should contribute to the discovery of new pharmacologically active compounds or new vector-exposure immunological markers while serving as a protein database for mass-spectrometric protein identification studies.

## Supporting information

S1 Spreadsheet Hyperlinked spreadsheet with protein and coding sequences obtained from the transcriptome assemblies. (XLSX)

S2 Spreadsheet Classified hyperlinked spreadsheet with worksheets containing salivary gland and fat body overexpressed gene products. (XLSX)

S1 Table List of transcripts and primer sequences used in Fig 7 experiment. (DOCX)
