# Supplementary material for: An insight into the salivary gland and fat body transcriptome of Panstrongylus lignarius (Hemiptera: Heteroptera), the main vector of Chagas disease in Peru
Source: PLoS Negl Trop Dis. 2018 Feb 20;12(2):e0006243. doi: 10.1371/journal.pntd.0006243 (PMC5834209; doi:10.1371/journal.pntd.0006243)
Supplement: S1 Table — (DOCX) [file pntd.0006243.s003.docx]

| INDEX | TRANSCRIPT | FUNCTION | SEQ. Forward | SEQ. Reverse |
| --- | --- | --- | --- | --- |
| 01 SG | PhSigP-59941_FR2_1-214 | Lipocalin | CAGCTATGGCAGGCTTTGAT | TCgACAgACAACAggTggAg |
| 02 SG | PhSigP-60467_FR2_11-1849 | Housekeeping - citoesq. | AATCACACCTgTTCCCCAAg | CCTCTCCgTTTgCTTgAATC |
| 03 SG | PhSigP-411_FR6_30-251 | Lipocalin | ggTTCAAAggAAgATggCAA | ACCTCggAACCATTACAACg |
| 04 SG | PhSigP-1017_FR6_104-314 | Lipocalin | TCACCTgAgAAACgAAgAACg | CATgCgTggCACACCTATAA |
| 05 SG | PhSigP-62745_FR6_1-235 | Protein syntesis machinery | TTCATgCCgATATgACTggA | ggACCgTTCAATgTCTCCAT |
| 06 SG | PhSigP-58976_FR6_1-213 | Lipocalin | TCgACAAAAAgAACCCAAgg | gCACgTAgCATCTTTTCTggA |
| 07 SG | PhSigP-7871_FR1_23-135 | Unknown | gCTTACCACATgCACCTCAA | TCgTgTgCAggTATTAgTggA |
| 08 SG | PhSigP-6876_FR4_1-138 | Conserved insect family 12 | ggTgACggATTgATCTTTCg | CCTTgggATTTgTCCTCTCA |
| 09 SG | PhSigP-60728_FR4_5-221 | Lipocalin | TgCATTTgCTCAAATTTCCA | CAAACgCATgTTCAATTTgg |
| 10 SG | PhSigP-56895_FR4_1-176 | Lipocalin | ATTgCAgTgCTgACAAggAA | TTTATAATgCAATTAggCggC |
| 11 SG | Ph-57329 | Housekeeping - citoesq. | AAATggTggTggCgAAAATA | CgCTTTCTTTgCCTTCATTC |
| 12 SG | Ph-59263 | Housekeeping - detoxif. | ATTATgggCCCTTTCgTCTC | CATTAACggTAgACTgCTgCC |
| 13 SG | Ph-58173 | Lipocalin | gCATTTCCAgTAgTCTgCCg | CCTgCTgCCAACACAAACTA |
| 14 SG | PhSigP-4154_FR5_1-147 | Conserved insect family 12 | AATTgCCgAAggTggTgTAg | TgCTggCTTCATACCgTAAA |
| 15 SG | PhSigP-62466_FR3_1-202 | Lipocalin | CggTCAgTTgTgAgggTACA | TCCggAACgATAATCTCTgC |
| 16 SG | PhSigP-51205_FR5_169-384 | Lipocalin | ATgTgCTTTTAAgCCgCAAT | ggCATTTCTgAgCTCgCTAC |
| 17 SG | PhSigP-60686 | Lipocalin | TgCTgATTATgCCACAgACC | TgggTCACgTACCAAgTACC |
| 18 SG | PhSigP-6695_FR4_1-225 | Hemiptera specific family 210 | AgTgCCgCCAAACTgTTTAC | CAgCTTTggCCAgTgTTTTT |
| 19 SG | PhSigP-1761_FR4_4-207 | Lipocalin | AggAACCgCCgTTAACAATA | TCCAgTCTTggAgCACCTTT |
| 20 SG | Ph-50672 | Lipocalin | gTCCgCTgAAACAggACCTA | CCATACTTTgCgTgTgTCgT |
| 01 FB | PhSigP-211_FR5_1-78 | Secreted protein | gCTCCTATTggCTggAACAA | CgAAgAAATTCCTgTgTgATgA |
| 02 FB | Ph-61586 | Unknown conserved | CCTCCgTgTCCAATATgTCC | ACCgTCCATTTgACCATCAC |
| 03 FB | PhSigP-57290_FR3_1-90 | Secreted protein | CCCTTCTCACTgTTTTTgCAg | ACAAATCggATCCCAAATCA |
| 04 FB | PhSigP-60586_FR5_1-72 | Secreted protein | CAATACAACAAgCCgAAgCA | TTgACACAggTgTgTgCTTgT |
| 05 FB | Ph-43785 | Amino acid metabolism | TCATCAgTgggATggggTAT | ATgTTTTCCCgTTCAATCCA |
| 06 FB | Ph-2737 | Cytoesqueletal protein | AAgCCgAATTATgCgAgATg | gAAACgTACCTTCAgggCAg |
| 07 FB | PhSigP-56177_FR2_1-105 | Secreted protein | CAAAAAgCCgATggACAAAT | ggCACTTTTAAATggCCCTA |
| 08 FB | Ph-51533 | Unknown conserved | TAgTCAAggATCCACCCCAg | gACAggCTTCCCTAgCTCCT |
| 09 FB | Ph-51416 | Signal transduction | TATTggCCAAAAgTgCTgCT | TgTTCgTTAAATACTgATggCCT |
| 10 FB | Ph-43262 | Amino acid metabolism | CCAATTggCCACCTTACACT | TCCAgCACAggTggTgAATA |
| 11 FB | Ph-62649 | Signal transduction | TgTTCgTCACCCgTTCACTA | AACCATATAATTCACCgCCg |
| 12 FB | Ph-2339 | Cytoeskeletal proteins | TgCCTTCCTCAAAgAACTgg | TTggAATTTTTgATgggTCC |
| 13 FB | Ph-5712 | Signal transduction | CgATCAATTCgTCATCCAAA | AAAgATCTCCACgTTCgCAT |
| 14 FB | Ph-563 | Unknown product | CgACCggAATTAgCAACAAT | TCCgAACCTggATCTggTAg |
| 15 FB | Ph-44680 | Protein modification | ggAAAAACCgTggAAgATCA | TggAATTCAACTTTggCTCC |
| 16 FB | Ph-55950 | Signal transduction | AACgTggCCCAgTTAAAgAA | CgTCACgATgCgTgATATTC |
| 17 FB | Ph-62759 | Lipid metabolism | TATgggACCAAATAAgCCgA | TgTACCggCATAAACAggTg |
| 18 FB | Ph-20238 | Transcription factor | ATAgCTCTggTTCgTgTgCC | AAAATCTggCCACCAAAATg |
| 19 FB | Ph-61290 | Intermediary metabolism | gTTgCAgTCCATTTTTCCgT | CgCATTgCTCggTATCTACA |
| 20 FB | Ph-59933 | Intermediary metabolism | ACATAgTgCATggTTTggCA | TTCTTgggAggCAAgAgTgT |
| 01 N | PhSigP-57396_FR2_1130-1211 | Secreted protein | CgTTTTCgCTTTTCCATCTg | CgACCTCgATgTTggATTTT |
| 02 N | Ph-2378 | Energy metabolism | gggTTTggAgATTATCCggT | TTTACTTggCCAAAAggggT |
| 03 N | PhSigP-2449_FR4_49-224 | Protein export | gCACTgCgAAACTACgAACA | CTCAgAgTTCAACCATggACC |
| 04 N | Ph-54498 | Amino acid metabolism | CCgTTgCTTggTTTgAAgAT | CATCgAATgggCTATgATCC |
| 05 N | Ph-57793 | Protein export | TCCAATgAATCCAgCAgTgA | TgggTAAACCCCATTTTgAg |
| 06 N | PhSigP-60851_FR5_2032-2148 | Unkown conserved membrane protein | TTgATCCCTCCgCTTACAgT | CATATgACTgAgCACCTgCg |
| 07 N | Ph-53366 | Unknown conserved | gCTTgCCCAgTTCCCATTTA | gCCCAgCTTgTAAAATAATggT |
| 08 N | Ph-1048 | Energy metabolism | ATTgCCATTTTgggTATggA | CTTggAAAggCTgAgACAgg |
| 09 N | Ph-47915 | Protein syntesis machinery | AggTACgATgTTCAAggTTTTTg | ACTTgAAgACCAggAggCAA |
| 10 N | Ph-5746 | Transcription machinery | TgCTgCTAAggTAgTTCgCC | TCCATCCCTCCAAggTgTAA |
| 11 N | Ph-61516 | Lipid metabolism | AACAAgCAACAACTggggAC | TTTTACCATTCCACgCTTCC |
| 12 N | PhSigP-59894_FR4_91-319 | Secreted protein | AgTTTgggAATTCgACATgg | TggTTTTCAgTTTTCCCACA |
| 13 N | Ph-56979 | Immunity | TgTgCTCTCCATggCACTAA | CAATggACCAATTCCCAATC |
| 14 N | PhSigP-51408_FR4_55-276 | Protein modification | TggAAAAATCATCAgTggCA | ATTCggCgATTACAACATCC |
| 15 N | Ph-638 | Transcription factor | CAAAAATgATCggATCTggC | TggTgTgTgCAATTCATCCT |
| 16 N | PhSigP-51836_FR6_21-331 | Protein modification | gACACCTCCTATCACCACCC | gACCACCCATTgTTCAggTT |
| 17 N | Ph-58308 | Cytoeskeletal proteins | TATATgggCCAAATCCgATg | TAACACCTgCCgATgTCAgA |
| 18 N | PhSigP-59044_FR3_1-125 | Secreted protein | ACAATAgCCgCTggTgAAAC | ATgTCgCCATCACAAACTgA |
| 19 N | Ph-1118 | Protein modification | CCAgAATTCgAAAAAgCTgC | ATTgCACgTTTCCTTTCCAC |
| 20 N | PhSigP-60789_FR6_18-581 | Secreted protein | AACTggTgCAgTTgAgggAg | TTATTgCCATAACCCCAggA |
| 21 N | PhSigP-44983_FR3_1-514 | Signal tranduction | CATCCTgggCggATTTATTA | TTTTgTgCCgAAAAggAgAT |
